# Multiple functions of stress granules in viral infection at a glance

**DOI:** 10.3389/fmicb.2023.1138864

**Published:** 2023-03-01

**Authors:** Yuelin Guan, Yan Wang, Xudong Fu, Guannan Bai, Xue Li, Jianhua Mao, Yongbin Yan, Lidan Hu

**Affiliations:** ^1^The Children’s Hospital, Zhejiang University School of Medicine, National Clinical Research Center for Child Health, Hangzhou, China; ^2^Center of Stem Cell and Regenerative Medicine, and Bone Marrow Transplantation Center of the First Affiliated Hospital, Zhejiang University School of Medicine, Hangzhou, Zhejiang, China; ^3^Zhejiang Laboratory for Systems and Precision Medicine, Zhejiang University Medical Center, Hangzhou, Zhejiang, China; ^4^Department of Big Data in Health Science School of Public Health and The Second Affiliated Hospital, Zhejiang University School of Medicine, Hangzhou, Zhejiang, China; ^5^State Key Laboratory of Membrane Biology, School of Life Sciences, Tsinghua University, Beijing, China

**Keywords:** stress granule, viral infection, SARS-CoV-2, drug design, antiviral drugs

## Abstract

Stress granules (SGs) are distinct RNA granules induced by various stresses, which are evolutionarily conserved across species. In general, SGs act as a conservative and essential self-protection mechanism during stress responses. Viruses have a long evolutionary history and viral infections can trigger a series of cellular stress responses, which may interact with SG formation. Targeting SGs is believed as one of the critical and conservative measures for viruses to tackle the inhibition of host cells. In this systematic review, we have summarized the role of SGs in viral infection and categorized their relationships into three tables, with a particular focus on Severe Acute Respiratory Syndrome Coronavirus 2 (SARS-CoV-2) infection. Moreover, we have outlined several kinds of drugs targeting SGs according to different pathways, most of which are potentially effective against SARS-CoV-2. We believe this review would offer a new view for the researchers and clinicians to attempt to develop more efficacious treatments for virus infection, particularly for the treatment of SARS-CoV-2 infection.

## 1. Introduction

In eukaryotic cells, the membraneless organelles composed of mRNA and proteins are called RNA granules (Anderson and Kedersha, 2009). Stress granules (SGs), one type of RNA granules, transiently form in the cytoplasm during cellular stress and are evolutionarily conservative in animals and plants (Spector, 2006; Reineke and Neilson, 2019). SGs are involved in the regulation of transcription and translation which is essential for maintaining cellular homeostasis. Life is full of transient stress, and eukaryotic cells have developed sophisticated coping mechanisms to deal with a bombardment of cellular challenges (Morimoto, 2011; Li et al., 2013). SG formation appears to be a prudent and essential mechanism during stress responses; it reduces energy use, restores cellular homeostasis, and increases cell viability under damaging conditions (Mahboubi and Stochaj, 2017).

SGs are composed of multiple factors including translation initiation factors, polyadenylated RNA, small ribosomal subunits, and numerous RNA binding proteins (RBPs; Thomas et al., 2011; Aulas and Vande Velde, 2015). These components can be divided into three grades (Fan and Leung, 2016). The innermost part exists in almost all SGs induced by various stress conditions. It consists of a 48S pre-initiation complex, along with stalled mRNA transcripts, such as poly (A)-binding protein-1 (PABP-1), eukaryotic initiation factor 3 (eIF3), eukaryotic initiation factor 4B (eIF4B), eukaryotic initiation factor 4F (eIF4F), and eukaryotic initiation factor 4A (eIF4A), etc., (Anderson and Kedersha, 2002). The middle part contains some scaffold proteins, such as GTPase-activating protein SH3 domain-binding protein 1/2 (G3BP1/2) and T cell intracellular antigen 1 (TIA-1; Matsuki et al., 2013). The outermost part contains variant signaling proteins based on the various cellular environment. Various amounts and sizes of SGs with specific stress-related components (i.e., protein and RNA) would be formed differently depending on the cell types, stress situations, and changes in action time (Moeller et al., 2004). In brief, as a rapid response signaling hub, SG with complex structures plays an important regulatory role in a variety of stress injuries. Along with this, it is reasonable to speculate that misregulated SG dynamics may induce an inaccurate cellular state of physiological activity of both RNA metabolism and protein homeostasis (Li et al., 2013; Portz et al., 2021).

Abnormal metabolism of SGs has been found in a variety of diseases, including but not limited to cancer, neurodegenerative diseases (NDs), viral infections, autoimmune disease, cataracts, glaucoma, diabetes, and brain ischemia (Moujaber et al., 2017). Given that SGs have drawn widespread concern in recent years, the correlations between cancer or NDs and SGs have already been widely described (Chen and Liu, 2017; Gao et al., 2019; Hu et al., 2022), while reviews about the role of SGs in viral infections are less understood. Some studies point out that viral infections can trigger a series of cellular stress reactions and consequently regulate the assembly or disassembly of SGs (McInerney et al., 2005), suggesting the importance of SGs in balancing the translation of host-and virus-encoded mRNAs (Reineke and Neilson, 2019; Eiermann et al., 2020). In this review, we mainly focus on recent advances in the correlation between viruses and SGs, which may provide insight into developing new effective antiviral treatments in clinical application.

To better understand the relationship between SGs and anti-virus, this review first briefly describes the background of SGs and the information about viruses. Based on the interactions between SG and viruses, viruses were categorized into three main groups, i.e., inhibition, promotion, and temporary promotion of SG formation. Secondly, this review further recapitulates the role of SGs in the regulation of antiviral response, especially for several important antiviral function pathways of SGs are also highlighted. In particular, given that Severe Acute Respiratory Syndrome Coronavirus 2 (SARS-CoV-2) has made the most far-reaching impact in the world since 2019, this review also summarizes the current evidence regarding the connection between SARS-CoV-2 and SGs, aiming to provide insights into developing novel SG-based drugs for clinical treatment of SARS-CoV-2 infection.

## 2. The dynamic processes of SGs

**Figure 1:**
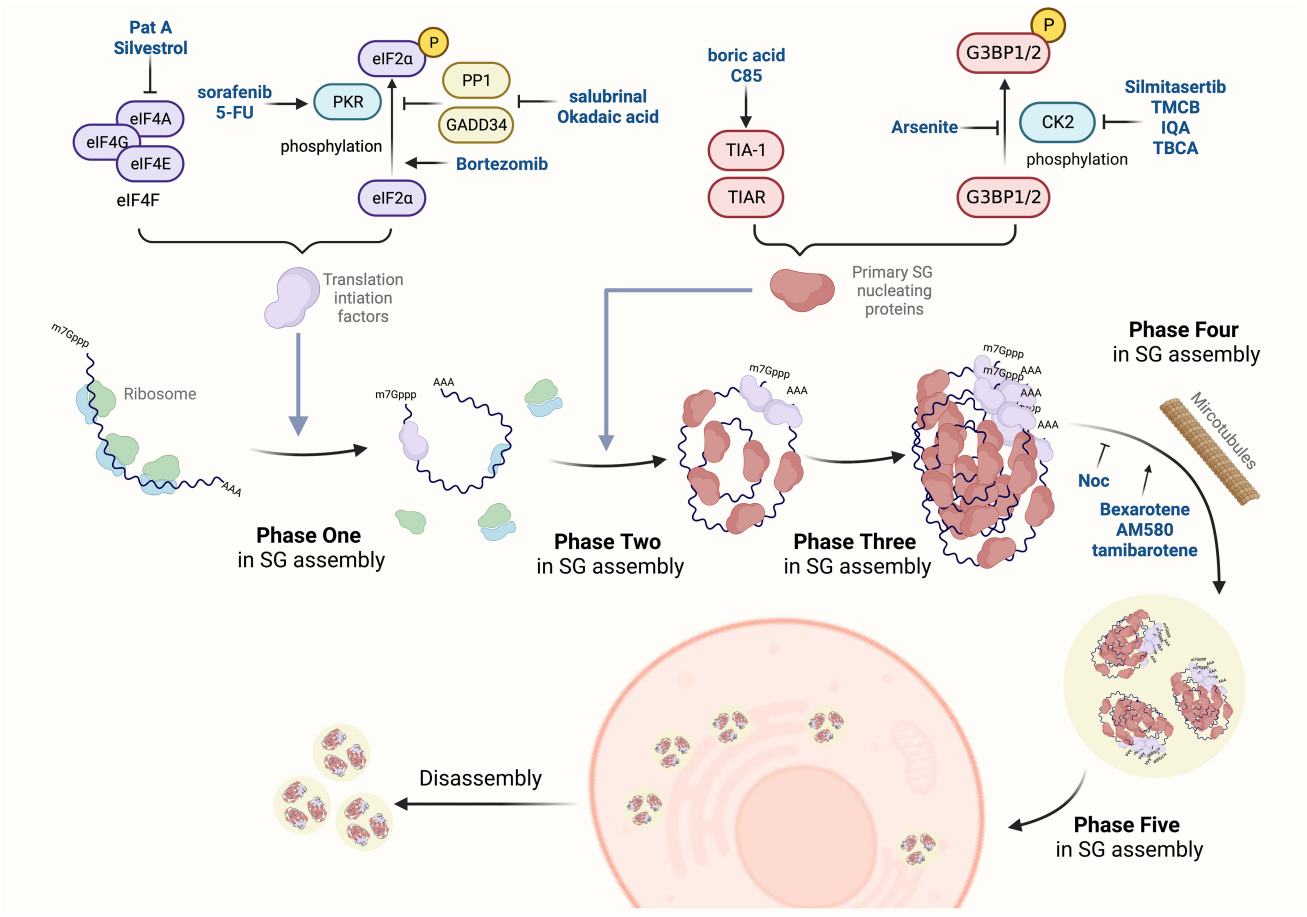
SGs-targeted antiviral drugs. The dynamic change of SGs is a complex process regulated by many post-translational modifications, protein remodeling complexes, and microtubule networks. The assembly of SG is divided into five phases. Phase one: SG assembly begins with stalled translation initiation, and ribosomes flow out to convert into mRNPs. Phase two: primary aggregation and nucleation occur when heterogeneous 48S-bound transcripts bind to self-aggregation RNA-binding proteins, such as G3BP1/2, TIA-1, tristetraprolin (TTP), and fragile X mental retardation protein (FMRP). Phase three: secondary aggregation and crosslinking occur when PABP-1 is bound to poly(A)-containing transcripts and smaller oligomers crosslink to assemble microscopic invisible aggregates. Phase four: some transcripts bind to multiple SG nucleating proteins, which enhance the cross-linking process to form progressively larger SGs, and then recruit non-RNA-binding proteins (e.g., TRAF2, plakophilins, SRC3, FAST). Phase five: specific transcripts are sorted out of SGs by translation initiation, assembling into other RNA granules. The disassembly of SGs is the reverse process. Novel drugs have been identified to affect the process of SGs assembly *via* different mechanisms.

SG formation appears to be a conservative and essential mechanism during stress responses. Once the cell recovers to its normal situation, SGs would transiently disassemble. The assembly and disassembly of SGs are regulated by environmental and physiological factors (Protter and Parker, 2016; Moujaber et al., 2017). By adjusting the balance between the translational repression mRNA and translating mRNAs, SG formation can handle the timely and appropriate response to stress conditions (Protter and Parker, 2016). The biogenesis of SGs under normal physiological conditions is usually divided into five phases based on the specific composition and localization of mRNPs (Anderson and Kedersha, 2008). Phase one: stalled initiation and ribosome runoff. Phase two: primary aggregation and nucleation. Phase three: secondary aggregation. Phase four: integration and signaling. Phase five: mRNA triage. SG disassembly is the reverse process. Large SGs are decomposed into small particles, and these small particles are subsequently depolymerized or removed (Kedersha et al., 2005). Along with the disappearance of stress, SGs are decomposed rapidly from the cytoplasm of cells through the chaperone pathway or autophagy pathway (Wheeler et al., 2016). These phases occur sequentially in normal conditions (the flow chart shown in Figure 1). Moreover, in some pathological conditions, such as hypoxia, a common feature of numerous pathological conditions, including myocardial infarction, stroke, inflammation, and malignant tumors, SG formation may inhibit cell apoptosis through translation arrest, prevention of unfolded proteins accumulation (Arimoto et al., 2008). Besides, SGs share many components with neuronal granules in neurons, which clearly indicates that SGs affect neurodegenerative diseases, amyotrophic lateral sclerosis (ALS) disease, frontotemporal lobar degeneration (FTLD), Alzheimer’s disease (AD) and Spinal muscular atrophy (SMA; Anderson et al., 2015; Brownsword and Locker, 2022; Hu et al., 2022).

Once SGs are absent in cells for some reasons, it may cause various abnormal physiological activities and even the occurrence of diseases (Gao et al., 2019). For instance, the deficiency of G3BP1 leads to abnormal synaptic plasticity, calcium homeostasis in neurons, and increases apoptotic cell death (Zekri et al., 2005; Martin et al., 2013). TIA-1 knockout (KO) mice worsen hepatic steatosis and fibrosis (Dolicka et al., 2022) and dysregulate expression of lipid storage and membrane dynamics factors in nervous tissue (Heck et al., 2014). In the case of viral infections, in G3BP1 KO cells, the replication efficiency of mammalian orthoreovirus (MRV) is significantly improved (Carroll et al., 2014). In murine TIA-1-related protein (TIAR) KO cells, West Nile virus (WNV) growth is decreased (Li et al., 2002). In the following texts, we will mainly focus on the relationship between SGs and viral infection.

## 3. Viral infection regulates the SG formation in double ended manners

During the process of viral infection, the assembly and disassembly of SGs are intensively regulated (McCormick and Khaperskyy, 2017). There has been evidence showing that viral infection could interfere with SG formation through various mechanisms (White and Lloyd, 2012), such as inhibiting translational initiation (Linero et al., 2011), sequestering SG components (such as TIA-1, and G3BP1/2) (Nikolic et al., 2016), and interacting with key SG proteins to form stable viral ribonucleoprotein (RNP) complexes (Abrahamyan et al., 2010). However, some viruses have developed mechanisms to blunt host responses and manipulate SGs to evade host defenses (Kim et al., 2016). Viruses can regulate SG formation by three major manners: inducing SG formation, inducing SG transient formation, or inhibiting SG formation. Protein kinase R (PKR), one of the major innate immune mechanisms, is the primary sensor responsible for host defense against invading viral pathogens *via* rapid inhibition of SG formation upon viral infection (Gao et al., 2022). And PKR is activated by double-stranded RNA (dsRNA) viruses. For the specific way of inducing SG transient formation, some RNA viruses activate the PKR pathway, resulting in the phosphorylation of eukaryotic initiator factor 2A (eIF2α) and promoting SG formation at the early stage of viral infection. Nevertheless, in the later stage of infection, they utilize several mechanisms to antagonize SG formation, such as G3BP1/2 cleavage and PKR inactivation to inhibit SG formation in turn (Ng et al., 2013; Okonski and Samuel, 2013). In the following text, we describe these three categories of viruses in detail.

### 3.1. Viruses induce SG formation

The first type of virus induces SG formation to aid viral RNA replication (Table 1). The “induced SGs” viruses are dedicated to activating eIF2α and/or recruiting SGs’ core components.

**Table 1:** Viruses induce SG formation.

Species	Type	Mechanism of formation	Refs
CSFV	(+) ssRNA	PKR phosphorylation, eIF2α phosphorylation	Liu et al. (2015)
HSV-2	dsDNA	Deletion of virion host shutoff protein (Vhs) inhibits elF2α phosphorylation	Finnen et al. (2014)
PRRSV	(+) ssRNA	eIF2α phosphorylation by PERK activation	Zhou et al. (2017)
PHEV	(+) ssRNA	activated PERK/PKR-eIF2α axis	Shi et al. (2022)
RSV	(−) ssRNA	PKR-mediated aggregation of SGs	Lindquist et al. (2011)
RVFV	(−) ssRNA	Down-regulation of PKB/mTOR signaling pathways and increased the activity of 4EBP1/2 proteins	Hopkins et al. (2015)
SINV	(+) ssRNA	Activation of GCN2 through its viral RNA; infection induces eIF2α phosphorylation, which leads to SG assembly	Jefferson et al. (2019)
TBEV	(+) ssRNA	Recruitment of TIA-1 and TIAR	Albornoz et al. (2014)
TGEV	(+) ssRNA	TIA-1/TIAR aggregation and elF2α phosphorylation	Sola et al. (2011)
VSV	(−) ssRNA	elF2α phosphorylation and SG-like particle formation and aggregation	Dinh et al. (2013)
VV	dsDNA	Deletion of E3L activates PKR	Simpson-Holley et al. (2011)

HSV-2, Herpes simplex virus type 2

Many viruses target the PKR/eIF2α pathway to trigger SG assembly and destroy the homeostasis of cells. For example, Sindbis virus (SINV) and Respiratory syncytial virus (RSV) infection activate PKR and induce eIF2α phosphorylation, which lead to SG assembly (Lindquist et al., 2011; Jefferson et al., 2019). Under oxidative stress and RSV infection, exposure to polyhexamethylene guanidine phosphate (PHMG-p) remarkably increases eIF2α phosphorylation and significantly increases SG formation (Choi et al., 2022). Given the limited space available, more targeted proteins of viruses are listed in Table 1. Beyond PKR, the virus can also activate other eIF2α kinases in cells. Porcine reproductive and respiratory syndrome virus (PRRSV) alternatively activates PKR-like endoplasmic reticulum kinase (PERK) to phosphorylate eIF2α and consequently stimulates cells to produce SGs (Zhou et al., 2017). Porcine hemagglutinating encephalomyelitis virus (PHEV) infection induces endoplasmic reticulum (ER) stress, activates the UPR, then activate PERK/PKR-eIF2α axis, as a result, promoting SG formation (Shi et al., 2022). Rift Valley fever virus (RVFV) reduces the PKB/mTOR (the protein kinase B, mechanistic target of rapamycin) signaling pathway, thereby increasing the activity of eIF4E binding protein 1/2 (4EBP1/2) to inhibit the translation process, and then cause transient SG assembly (Hopkins et al., 2015). Vesicular stomatitis virus (VSV) infection of host cells will induce elF2α phosphorylation and promote SG-like particle formation and assembly (Dinh et al., 2013).

Besides, TIA-1/TIAR can be recruited to their replication sites to form SGs when host cells are infected by tick-borne encephalitis virus (TBEV) (Albornoz et al., 2014). Porcine transmissible gastroenteritis virus (TGEV) infection will also induce TIA-1/TIAR aggregation and elF2α phosphorylation, resulting in SG assembly at the late stage (Sola et al., 2011). Given that the regulation of SG formation is crucial for the replication of infected viruses, drugs that inhibit SGs by bypassing PKR (or other kinases) and/or eIF2α phosphorylation may have therapeutic potential to control the virus replication.

### 3.2. Viruses trigger SG formation temporarily

The second type of virus is featured to temporarily trigger SG formation in the early replication cycle but limit SG formation in the late replication cycle (Table 2).

**Table 2:** Viruses trigger SG formation temporarily.

Species	Type	Mechanism of formation	Refs
HCV	(+) ssRNA	Phosphorylation level of elF2α determines SG formation and depolymerization	Ruggieri et al. (2012)
MHV	(+) ssRNA	Addition of eIF2α phosphorylation	Raaben et al. (2007)
MRV	dsRNA	The high phosphorylation level of elF2α and interaction between G3BP1 and μNS	Qin et al. (2009), Qin et al. (2011) and Carroll et al. (2014)
PEDV	(+) ssRNA	Caspase-8-mediated cleavage of G3BP1 inhibits SG assembly and SG assembly is impaired by silencing G3BP1	Sun et al. (2021) and Guo et al. (2022)
PV	(+) ssRNA	Protease 2A can induce the generation of SGs at first, and the cleavage of G3BP1 by PV3C protease leads to SG depolymerization later	Dougherty et al. (2015) and Yang et al. (2018)
WNV	(+) ssRNA	W956IC can efficiently induce SGs through PKR activation	Courtney et al. (2012)

The vast majority of these viruses regulate SG dynamics by regulating elF2α phosphorylation. Hepatitis C virus (HCV) infection rapidly induces the production of SGs in the early stage, and then the depolymerization of SGs occurs later, and this change happens depending on the phosphorylation level of elF2α (Ruggieri et al., 2012). In the early stage of infection, mouse hepatitis coronavirus (MHV) causes SG formation by promoting the elF2α phosphorylation (Raaben et al., 2007). MRV induces SG formation (Qin et al., 2009) in the early stage of infection, but the SG formation is reduced in the late stage of infection, regardless of the high elF2α phosphorylation (Qin et al., 2011). Although natural WNV infection does not induce SGs, the W956IC (a lineage 2/1 chimeric WNV infectious clone) efficiently induces SGs through PKR activation to phosphorylate elF2α at the early infection stage (Courtney et al., 2012).

Overexpression of G3BP may induce spontaneous SG formation (White and Lloyd, 2012). It has also been reported that MRV can recruit the viral non-structural protein μNS to interact with G3BP1, which interferes with SG assembly (Carroll et al., 2014). Epidemic diarrhea virus (PEDV) infection results in the cleavage of G3BP1 and this process is mediated by caspase-8 (Sun et al., 2021). And PEDV replication is significantly enhanced when SG assembly is impaired by silencing G3BP1 (Guo et al., 2022). Besides, protease 2A of poliovirus (PV) induces the generation of SGs at first, and the cleavage of G3BP1 by PV3C protease leads to SG disassembly later (Yang et al., 2018).

### 3.3. Viruses inhibit SG formation

Contrary to the above mechanisms, a prevalent group of viruses impinges on SG formation throughout the process of infection in Table 3.

**Table 3:** Viruses inhibit SG formation.

Species	Type	Mechanism of inhibition	Refs
CHIKV	(+) ssRNA	GADD34 to enhance the dephosphorylation of elF2α	Clavarino et al. (2012)
CVB3	(+) ssRNA	G3BP1 cleavage	Fung et al. (2013)
DENV	(+) ssRNA	recruit TIA/TIAR to replication complexes	Emara and Brinton (2007)
EBOV	(−) ssRNA	Inhibition of PKR pathway by VP35	Le Sage et al. (2017)
EMCV	(+) ssRNA	G3BP1 cleavage	Ng et al. (2013)
FCV	(+) ssRNA	The viral protease NSP6 to cleave G3BP1	Humoud et al. (2016)
FMDV	(+) ssRNA	L protein to stably interact with G3BP1	Visser et al. (2019)
HCMV	dsDNA	Encodement of pTRS1 to interact with PKR	Vincent et al. (2017)
HIV-1	ssRNA-RT	Assembly of SHRNP	Abrahamyan et al. (2010)
HSV	dsDNA	Inhibition of PERK activation and elF2α phosphorylation by surface glycoprotein gB; UL41 interferes with SG formation through its endoribonuclease activity	Mulvey et al. (2007) and Finnen et al. (2016)
HTLV-1	ssRNA-RT	Interaction with HDAC6 through Tax	Legros et al. (2011)
IAV	(−) ssRNA	Inhibition of PKR activation by NS1; Regulation of SGs assembly by DDX3X	Khaperskyy et al. (2012), Khaperskyy et al. (2014) and Kesavardhana et al. (2021)
IBV	(+) ssRNA	Inhibition of PKR activation through NSP2；Up-regulation of GADD34；Increase dephosphorylated activity of PP1	Wang et al. (2009) and Burgess and Mohr (2018)
JEV	(+) ssRNA	G3BP1 isolation and interaction with CAPRIN1	Katoh et al. (2013)
KSHV	dsDNA	Expression of ORF57 to bind to PKR	Sharma et al. (2017) and Sharma and Zheng (2021)
Mengovirus	(+) ssRNA	PKR-dependent aggregation of G3BP1 by L protein	Reineke et al. (2015)
MERS-CoV	(+) ssRNA	Inhibition of PKR-mediated elF2α phosphorylation	Nakagawa et al. (2018)
MeV	(−) ssRNA	Inhibition of PKR-dependent SG aggregation by protein-C	Randall and Goodbourn (2008)
PRV	dsRNA	Dephosphorylation of elF2α	Xu et al. (2020)
Rotavirus	dsRNA	Block the PKR-eIF2α phosphorylation; Transferation of PABP from cytoplasm to nucleus	Montero et al. (2008), Lopez and Arias (2012) and Dhillon and Rao (2018)
SARS-CoV-2	(+) ssRNA	The N protein attenuates SG formation by localizing to the SGs and sequestering G3BP1/2 from their interacting proteins	Bouhaddou et al. (2020) and Gordon et al. (2020b)
SEV	ssRNA-RT	The transcription product interacts with TIAR to inhibit the generation of SGs	Iseni et al. (2002)
TMEV	(+) ssRNA	Expression of leader (L) protein to stably interact with G3BP1	Borghese and Michiels (2011)
WNV	(+) ssRNA	Interaction with TIA-1 and TIAR to inhibit SG formation	Li et al. (2002) and Emara and Brinton (2007)
ZIKV	(+) ssRNA	Hijack of G3BP1 and CAPRIN1	Hou et al. (2017)

Some viruses encode PKR inhibitors, thereby avoiding PKR-mediated phosphorylation of eIF2α and SG formation (Malinowska et al., 2016). Given the limited space available, only some mediate proteins of viruses were described in detail, and we have listed more target proteins of viruses in Table 3. For example, influenza A virus (IAV) and infectious bronchitis virus (IBV) inhibit the activity of PKR to block the phosphorylation of eIF2α by the non-structural protein 1 (NSP1) and non-structural protein 2 (NSP2), respectively, which in turn inhibits SG formation (Khaperskyy et al., 2014; Burgess and Mohr, 2018). Intriguingly, Middle East respiratory syndrome Coronavirus (MERS-CoV) inhibits the SG formation by inhibiting PKR-mediated elF2α phosphorylation, while lacking subunits 4a and 4b MERS-CoV induces the SG formation (Nakagawa et al., 2018). For rotavirus-infected host cells, it blocks host protein synthesis, PKR activation, eIF2α phosphorylation, and modification of cellular translation machinery (Lopez and Arias, 2012). Beyond the PKR pathway, there are other pathways, e.g., PERK pathway, regulating the phosphorylation of eIF2α. Herpes simplex virus (HSV), as a dsDNA virus, inhibits the activation of PERK and hinders the eIF2α phosphorylation through the surface glycoprotein B (Mulvey et al., 2007). In addition, several other viruses regulate the eIF2α dephosphorylation. Chikungunya virus (CHIKV) induces the expression of DNA-damage-inducible 34 (GADD34) to increase the dephosphorylation of eIF2α (Clavarino et al., 2012). And Pseudorabies virus (PRV) infection significantly inhibits the SG formation by dephosphorylating eIF2α, such as Chikungunya virus (CHIKV) and Pseudorabies virus (PRV; Xu et al., 2020).

Moreover, except for regulating the phosphorylation of eIF2α, viruses can inhibit SG formation *via* interaction with SG components, especially the scaffold proteins, e.g., G3BP1 and TIA-1. For example, Theiler’s murine encephalomyelitis virus (TMEV) and foot-and-mouth disease virus (FMDV; Visser et al., 2019) interfere with SG formation by leader (L) protein to stably sequester G3BP1. Similarity, the Japanese encephalitis virus (JEV) and Zika virus (ZIKV) sequester G3BP1 by interacting with the cell cycle-associated protein 1 (CAPRIN1; Hou et al., 2017). Moreover, feline calicivirus (FCV), encephalomyocarditis virus (EMCV; Ng et al., 2013), and coxsackievirus B3 (CVB3; Fung et al., 2013) can produce the viral protease to cleave G3BP1, thereby disrupting the assembly of SGs (Humoud et al., 2016). In addition to G3BP1, TIA-1/TIAR are also targeted by viruses to interfere with SG formation. The 3′ stem-loop structure in WNV, Sendai virus’s (SEV) transcription product, and dengue virus (DENV) could interact with TIA/TIAR which inhibit SG formation (Li et al., 2002; Emara and Brinton, 2007). Beyond scaffold proteins, other SG core components are also involved in viral infection (Iseni et al., 2002; Emara and Brinton, 2007; more detail in Table 1). Human immunodeficiency virus type 1 (HIV-1) significantly inhibits SG formation by assembling the Staufen1-containing HIV-1-dependent ribonucleoproteins (SHRNP) in host cells (Abrahamyan et al., 2010). Moreover, the host DEAD-box box helicase 3X-linked protein (DDX3X) also coordinates various antiviral responses in IAV infection, including regulation of SG assembly (Kesavardhana et al., 2021). Taken together, the core components of SGs (G3BP1, TIA-1/TIAR, HDAC6, SHRNP, DDX3X, GADD34, PP1) can be regulated by viruses to eventually affect SG formation.

It is worth mentioning that SARS-CoV-2 can also inhibit SG formation. Given the global pandemic caused by SARS-CoV-2, this review makes effort to elaborate on the interactive relationship between SARS-CoV-2 and SGs. SARS-CoV-2, a positive-sense single-stranded RNA (ssRNA) virus (Zhang et al., 2019), includes 30 kb of genomic RNA and four structural proteins (the crown spike (S) glycoprotein, the membrane (M) protein, ion channels envelope (E) protein, and nucleocapsid (N) protein; Wang Q. et al., 2020). And post-translational modifications (PTMs) related to SARS-CoV-2, like glycosylation and phosphorylation, are also pathogenic (Cheng et al., 2022). The S protein consists of two subunits, S1 and S2, which play a key role in receptor recognition and virus-cell membrane fusion. The glycosylation of SARS-CoV-2 mainly occurs on the S protein, which mediates the interaction with cellular receptors angiotensin-converting enzyme 2 (ACE2). After binding to ACE2, the S protein would alter its conformation, then resulting in viral membrane fusion (Zhou et al., 2020). As for the N protein, it has two distinct RNA-binding domains, involved in multiple aspects of the viral life cycle, including viral genomic RNA replication and virion assembly. The RNA intercalator mitoxantrone disrupts N protein assembly *in vitro* and in cells (Somasekharan and Gleave, 2021). Furthermore, the N protein is highly produced in infected cells to increase the efficiency of subgenomic viral RNA transcription, regulate host cell metabolism (Liu W. et al., 2020), and mediate the suppression of host antiviral responses (Nabeel-Shah et al., 2022; Wang et al., 2022). The interaction between the N protein and G3BP1/2 supports SARS-CoV-2 infection. Some studies agree that the N protein could disrupt SG formation by sequestering G3BP1/2 from interacting with other proteins (Stukalov et al., 2021; Kim et al., 2022). The non-structural protein 1 (Nsp1) of the virus can decrease the level of G3BP1, which is associated with nuclear accumulation of the SG-nucleating protein TIAR (Dolliver et al., 2022). Besides, methyltransferases 1 (PRMT1) methylates SARS-CoV-2 N protein at residues R95 and R177. It is reported that the methylation of R95 can regulate the ability of N protein to suppress SG formation (Cai T. et al., 2021). Meanwhile, the phosphorylation of the N protein can also interfere with the SG formation (Cheng et al., 2022). For instance, the inhibition of SG formation by SARS-CoV-2 may be mediated through the interaction of N protein with casein kinase 2 (CK2) subunits, like G3BP1/2, casein kinase 2 beta/casein kinase 2 alpha 2 (CSNK2B/CSNK2A2; Gordon et al., 2020b).

To summarize, viruses have evolved several mechanisms to counteract the restrictive effect of translational repression. Some viruses, mainly ssRNA viruses, replicate by inducing or controlling SG formation (Tables 1, 2). Other viruses achieve efficient replication by preventing SG formation *via* a variety of mechanisms. This strategy is the most popular choice for viruses, including ssRNA viruses, dsRNA viruses, dsDNA viruses, and retroviruses (Table 3).

## 4. The antiviral effect of SGs

As described above, SGs can interact with virus replication *via* multiple mechanisms, which might be promising targets for antiviral intervention. Given that SARS-CoV-2 belongs to the type of virus that inhibits SG formation, in this section, we outline the reported small molecules that can trigger SG formation and discuss the prospects for developing antiviral drugs (Figure 1).

### 4.1. The pathways involved in anti-virus

It is generally believed that SG formation can affect translation, which will inhibit viral replication (Nikolic et al., 2016). The translation of some viruses is strictly dependent on the 40S subunit and eIF4G, and these translation initiation factors are retained in SGs, which is not conducive to the translation of viral proteins (Liu Y. et al., 2020). PKR and PERK are the two enzymes related to translation and PKR activation during certain viral infections. Meanwhile, the assembly of the viral replication complex is affected when G3BP1 or TIA-1/TIAR remains in the SG, (Fritzlar et al., 2019). For instance, the 3′ terminal neck structure of WNV, TBEV, ZIKV, and JEV can interact with TIA-1/TIAR to regulate viral replication (Bonenfant et al., 2019). Some viruses like Vaccinia virus (VV), MRV, and DENV recruit G3BP1 to assist the replication of viruses around the viral replication complex. The RNA recognition receptor retinoic acid-inducible gene-I (RIG-I) is retained in SG and activated by dsRNA in SG to activate the innate immune response of cells (McInerney et al., 2005). In both human and mouse cells, the deletion of G3BP1 leads to insufficient binding of RNA by RIG-I (Cai H. et al., 2021). In conclusion, the cell can sense the virus from multiple aspects, inhibit its translation, and resist viral infection. It is the SG that can provide a platform for the recognition of pathogene-related molecular patterns, activates the immune signaling pathway of host cells. Therefore, SGs are generally considered to have antiviral effects upon viral infection (Yang et al., 2019; Zhang et al., 2019).

### 4.2. SG-targeted antiviral small molecules

#### 4.2.1. miRNAs targeted gene coded SG-associated protein

Previous studies have found that miRNAs can be used as targeting SGs. Three miRNAs have been reported, hsa-miR-615-3p, hsa-miR-221-3p, and hsa-miR-124-3p, which target at least two of the five key genes coded SG scaffold proteins (Prasad et al., 2021). One of the studies have shown that mitogen-activated protein kinase-activated protein kinase 2 (MAPKAPK2) in the lungs of SARS-CoV-2 patients could be reduced by hsa-miR-615-3p (Jafarinejad-Farsangi et al., 2020). The hsa-miR-221-3p, which targets ADAM17 (a disintegrin and metallo protease 17), is upregulated in hamster lung tissue infected by SARS-CoV-2 (Kim et al., 2020). It has been shown that SARS-CoV-2 hijacks DEAD box polypeptide 58 (DDX58), but hsa-miR-124-3p binds to DDX58 and inhibits SARS-CoV-2 genome replication eventually (Arora et al., 2020). Besides, hsa-miR-124-3p is found to be down-regulated in JEV-infected human neural stem cells (Mukherjee et al., 2019) and reduced pro-inflammatory cytokines Interleukin 6 (IL-6) and tumor necrosis factor alpha (TNFα) to prevent lung injury (Liang et al., 2020).

#### 4.2.2. Compounds targeted phase one in SG assembly

The major signaling pathways that regulate SG formation include the eIF2α and eIF4F pathways, and mTOR. As we mentioned in the background, PKR and eIF2α kinases are responsible for SG formation under different stresses, which provide effective drug targets for therapeutic intervention (Wang F. et al., 2020). Several small molecules have been reported to induce eIF2α phosphorylation. The RAF1/MEK/ERK kinase (Rubisco assembly factor 1, mitogen-activated protein kinase kinase, extracellular signal-regulated kinase) inhibitor sorafenib (Abdelgalil et al., 2019) and the anti-tumor drug 5-fluorouracil (5-FU; Kaehler et al., 2014) have been found to induce SGs assembly, inhibit cell proliferation and promote apoptosis *via* PKR-mediated eIF2α phosphorylation. Bortezomib, a peptide boronate inhibitor, efficiently induces SGs in many cancer cells and eIF2α phosphorylation.

In addition, PP1 and GADD34 are induced by phosphorylated eIF2α, and GADD34 provides negative feedback on eIF2α phosphorylation (Walter and Ron, 2011). Okadaic acid and salubrinal are well-known PP1 inhibitors (Nakagawa et al., 2018). These two chemicals may interfere with the interaction between PP1 and GADD34 and prevent eIF2α dephosphorylation. Okadaic acid is another well-known PP1 inhibitor (Nakagawa et al., 2018). It has been reported that viruses interfere with SG formation through the dephosphorylation of eIF2α by PP1 and GADD34 (Fusade-Boyer et al., 2019). These two chemicals may interfere with the interaction between PP1 and GADD34 and prevent eIF2α dephosphorylation.

The key eIF4F cap-binding complex components (eIF4A, eIF4E, and eIF4G) also mediate SG formation, which are candidates for coronavirus therapeutic targets. The promotion of G3BP aggregation by eIF4A inhibitors may partly explain their antiviral activities (Gordon et al., 2020a). Pateamine A (PatA) and silvestrol are natural products that disrupt eIF4A function and prevent translation, resulting in SG formation. Studies have shown that inhibition of SGs by Silvestrol affects the synthesis and replication of IAV protein (Slaine et al., 2017). Treatment of early viral infection by PatA and silvestrol will promote SG formation, arrest viral protein synthesis, and lead to failure of viral genome replication. PatA binds irreversibly to eIF4A, blocks IAV replication long-term after discontinuation, and inhibits IAV replication. In contrast, the antiviral effect of silvestrol is fully reversible, leading to rapid SG clearance and recovery of viral protein synthesis upon discontinuation. This study supports the feasibility of targeting the core host protein synthesis machinery to prevent viral replication (Slaine et al., 2017).

#### 4.2.3. Compounds targeted phase two in SG assembly

Targeting SG components may influence the dynamics of SGs. Particularly, G3BP1/2 and TIA-1 are essential for the initiation of SG formation. Small molecules targeting these proteins have the potential for antiviral therapy.

G3BP1/2 contains RNA-binding domains to assist RNA binding. Many viruses affect SG formation through G3BP1/2. Arsenite induces SG formation, probably *via* inducing the dephosphorylation of G3BP1/2 at Ser149 (Gallouzi et al., 1998). CK2 accelerates SG disassembly by promoting G3BP1 phosphorylation (Reineke et al., 2017). Silmitasertib, a CK2 inhibitor, inhibits CK2 and promotes SG formation, showing potent antiviral activity (Ahamad et al., 2020; Gordon et al., 2020a). Clinical trials of silmitasertib as a potential drug for SARS-CoV-2 treatment are currently under consideration (Yadav et al., 2022), suggesting that CK2 plays a role in regulating the SARS-CoV-2 life cycle. Similar to silmitasertib, TMCB also interferes with the disassembly of SGs by targeting CK2 and interacting with the carboxy-terminal domain (Ahamad et al., 2020; Wang F. et al., 2020). Besides, the cells pre-treated with CK2 inhibitor 5-oxo-5,6-dihydroindolo-(1,2-a) quinazoline-7-yl acetic acid (IQA) generates 2.5-fold SG production after Mengo virus with mutant L protein (Mengo-Zn) infection (Langereis et al., 2013) and cannot decompose SGs (Reineke et al., 2015). It is suggested that IQA can inhibit virus-induced SG breakdown (Reineke et al., 2017). Tetrabromocinna mic acid (TBCA) is also a specific CK2 inhibitor. TBCA treatment alone neither alters nor induces SG formation, but residual SGs in cells are increased under arsenite stress (Reineke et al., 2017). This feature has become a new strategy for TBCA to combine other drugs to fight viral infection.

Usually, as a scaffold protein of SG, TIA-1 is an RNA-binding protein and is associated with RNA and other proteins to form SGs *in vivo*. Interaction of TIA-1/TIAR with WNV, ZIKV, TBEV, PV, and DENV products in infected cells interferes with SG formation (Emara and Brinton, 2007; White and Lloyd, 2011; Albornoz et al., 2014; Bonenfant et al., 2019). Boric acid also balances the anti-apoptotic eIF2α-SGs pathway and pro-apoptotic pathway *via* promoting TIA-1 translocation from the nucleus to SGs (Henderson et al., 2015). Moreover, C85 (troxerutin) is very effective for SG formation induced by TIA-1 overexpression or arsenite treatment (Hu et al., 2017). Even more, it has been approved for human therapeutic usage by FDA and found to act as a SARS-CoV-2 main protease inhibitor, representing potential treatment options (Farhat and Khan, 2021). Particularly, C85 could stabilize SGs and perturb the equilibrium between reversible SG assembly and disassembly.

#### 4.2.4. Compounds targeted phase four in SG assembly

Microtubules are intracellular structures involved in the biological processes of cell division, organization of intracellular structures, and intracellular transportation. Microtubule disruption would delay SG formation, in which, as a consequence, SGs are formed smaller in size, greater in number, and variable in distribution (Nadezhdina et al., 2010). Based on enrichment analysis, Bexarotene (also known as targretin) has been found to upregulate the expression of SG proteins (i.e., DYNC1H1, DCTN1, and LMNA) in rats (Prasad et al., 2021). These proteins are associated with microtubules. Recently, Yuan et al. have shown that Bexarotene effectively inhibited SARS-CoV-2 replication *in vitro* (Yuan et al., 2020). It has been previously shown that AM580 and tamibarotene belong to the same drug class as Bexarotene, showing broad-spectrum antiviral activity against influenza virus, enterovirus A71, Zika virus, adenovirus, MERS-CoV and SARS-CoV (Yuan et al., 2019). Moreover, NDV infection induces canonical SGs and relatively small round granules are formed after treatment with nocodazole (Noc), a microtubule-disrupting drug. Unlike the large and irregular SGs in NDV-infected cells, Noc treatment induces marked microtubule depolymerization, inducing the formation of small, round granules (Sun et al., 2017). Taking current findings together, compounds targeting the protein elements of SGs have the promising potential for antiviral effect on SARS-CoV-2 infection.

## 5. Conclusion

In this review, we provide an overview of the composition, function, dynamic regulation, and viral-related mechanisms of SGs to help understand the role of SGs in viral infection. We then focus on the regulating function of SGs in the context of viruses, in particular the PKR-elF2α pathway, *via* which many viruses induce or inhibit SG formation by directly affecting elF2α phosphorylation. Specifically, we depict the interaction between SARS-CoV-2 and SG. We believed that several small molecules, including some inhibitors disrupting the interaction of G3BP1/2 with N protein, PRMT inhibitors, and CK2 inhibitors, could be considered as new therapeutic targets against SARS-CoV-2 infection *via* the regulation of SG assembly and dynamics. We also summarize potential antiviral drugs targeting on SGs, including small molecule compounds, such as Salubrinal, Okadaic acid PatA, silvestrol, and Noc. Finally, we describe the mechanism of anti-SARS-CoV-2, including Silmitasertib, TMCB, Bexarotene, and three miRNAs. Overall, our review summarizes the antiviral mechanisms of SGs and provides new insights into the development of SG-targeted antiviral drugs, particularly, the potential drugs against SARS-CoV-2.

## Author contributions

LH, YY, and YG conceived, designed, and supervised the research. YG wrote the manuscript and draw the figure. YW wrote the manuscript and categorized the tables. XF, GB, XL, and JM proofread and polished the manuscript. All authors contributed to the article and approved the submitted version.

## Funding

This study was financially supported by LH by the grant of the Natural Science Foundation of Zhejiang Province (no. LQ22C070004) and the Administration of Tranditional Chinese Mdeicine of Zhejiang Province (no. GZY-ZJ-KJ-23083).

## Conflict of interest

The authors declare that the research was conducted in the absence of any commercial or financial relationships that could be construed as a potential conflict of interest.

## Publisher’s note

All claims expressed in this article are solely those of the authors and do not necessarily represent those of their affiliated organizations, or those of the publisher, the editors and the reviewers. Any product that may be evaluated in this article, or claim that may be made by its manufacturer, is not guaranteed or endorsed by the publisher.
